# Reform of the first year of medical studies and diversification of student profiles in France: an unmet need?

**DOI:** 10.1186/s12909-024-05570-4

**Published:** 2024-05-28

**Authors:** Raoul K. Khanna, Emmanuelle Blanchard, Jeremy Pasco, Patrice Diot, Denis Angoulvant

**Affiliations:** 1 Department of ophthalmology, University Hospital of Tours, INSERM 1253 iBraiN, Tours, France; 2 Department of medical pedagogy, Faculty of medicine, University Hospital of Tours, 2 boulevard Tonnellé, Tours, 37000 France; 3 Unité de Recherche Clinique & Innovation, Centre Hospitalier Public du Cotentin, Cherbourg, France

**Keywords:** Medicine, Pedagogy, Undergraduate medical education, Diversification

## Abstract

**Objectives:**

To determine whether the reform of the first year of medical studies implemented in September 2020 in France met its objective of diversifying the profiles of students admitted to second year at the faculty of medicine at the University of Tours.

**Methods:**

Single-centered, retrospective study, covering students who passed the first year of medical studies between 2018 and 2022. Student profiles originating from three different entry gateways (PACES, PASS and L.AS) to the second year of medical studies were compared.

**Results:**

One thousand four hundred and seventy-nine students over five promotions were included (806 in PACES, 329 in PASS, 198 in L.AS). The ratio of students who had obtained a baccalaureate with high or highest honors was significantly higher in PACES (85%) and PASS (96%) compared to L.AS (66%; *p *< 0.001). These differences were related to increased student intake via a standard pass in L.AS (21% compared to 3.2% in PACES and 0.9% in PASS) (*p <* 0.001). In terms of geographical origin, the proportion of students residing in regions outside the University City area increased significantly in L.AS (11%) compared to PACES (1.7%) and PASS (3.3%) (*p* < 0.001). The mean number of parents from the white-collar and knowledge professional category was significantly higher in PACES (0.91) and PASS (1.06) compared to L.AS (0.80; *p <* 0.001).

**Conclusion:**

Students with a scientific background and who obtained highest honors in their high school diploma, remain the standard in PACES and PASS. Diversification of student profiles was achieved only within the L.AS gateway, which represented 42% of total second year admissions during the post-reform year. Student profile diversification was therefore a partially achieved objective and follow up studies of future promotions is needed to assess the medium and long-term impact of the reform. Particular attention should be paid to the future of these students who have different profiles between L.AS and PASS to determine whether these changes will have any impact in the quality of healthcare for the French population.

**Supplementary Information:**

The online version contains supplementary material available at 10.1186/s12909-024-05570-4.

## Introduction

The medical education system in France has undergone a significant overhaul over the past decade across all three cycles of study, from revising the competitive entry exam to reorganizing phased professionalization. The changes in the first cycle, implemented in 2020, aimed to broaden access, with the goal of enhancing student diversity across various socioeconomic backgrounds. In the second cycle, the focus shifted from purely imparting knowledge to facilitating skill acquisition, a change introduced in 2020. The third cycle introduced the challenge of promoting professionalization through three consecutive sequences.

Before the recent changes, from 2010 to 2020, entry into medical studies in France was secured through a competitive selection exam held during the First Common Core Year for Health Studies (PACES). This exam was prepared for during one academic year and served as an essential prerequisite for various healthcare disciplines, including medicine, midwifery, dentistry, pharmacy, and physiotherapy. Students had two opportunities to take this exam: their initial attempt and a single re-sit, if necessary. Success was determined by their PACES ranking; high-ranking students progressed to the second year of their chosen course, while others who did not succeed had the option of one re-sit.

The PACES pathway has been criticized for its elitist approach. Candidates failing to achieve the required cutoff (up to 40%) found that their year of study yielded no educational credits transferable to subsequent courses [1, 2]. In 2020, the PACES was replaced by a double entry pathway, i.e., the Health Access Specific Pathway (PASS) and a Bachelor’s degree with Health Access minor (L.AS) (Table 1).

**Table 1:** Characteristics of the three entry pathways to medical studies

	Specificities	Summary
**First Common year of Health Studies (PACES)**	Older system for entering health studies. One-year program followed by a competitive exam for medical, dental, pharmacy, or midwifery school with an opportunity to repeat the year in case of failure.	This system was highly selective, with a large number of students competing for a limited number of posts.
**Health Access Specific Pathway** **(PASS)**	Replaced PACES. One-year program with health subjects plus another disciplinary field (e.g., psychology). Competitive entry into medical studies with no possibility of repeating the year.	Not all students in PASS aim for medical studies. Those who do not pass or choose not to continue in the medical studies can pursue other academic paths using the disciplinary field they studied alongside health sciences.
**Bachelor degree with Health Access Minor (L.AS)**	Bachelor’s degree with a health option alongside another major (e.g., mathematics). The best-ranked students can apply for medical studies.	This system allows students to receive a more comprehensive educational background and is believed to be less stressful, as the environment and selection are less competitive.

The first aim of the medical studies admissions reform was to widen the admissions pathways to increase student diversity and opportunities, based on the hypothesis that a more inclusive student typology might improve the variety of future medical doctors (MDs), potentially counteracting unequal healthcare access in France [3]. Additionally, the reform required students to choose a mandatory minor discipline, offering an alternative to traditional medical studies. The reform also aimed to reduce the number of students facing academic failure by implementing a forward-moving system, thereby eliminating the need to repeat a year.

The current study aimed to evaluate the impact of the reform on student typology, based on the data of students who passed the first year of medical studies at the University of Tours, France, before and after the reform of the first year of medical studies.

## Methods

### Design

This retrospective study included second-year medical students at the University of Tours, France, who successfully completed the first common core year (PACES and then PASS or L.AS). The study spanned from September 2018 to September 2023 and covered the academic years 2018–2019, 2019–2020, 2020–2021, 2021–2022, and 2022–2023. The reform, implemented in 2020 for first-year students, consequently affected the 2021–2022 and 2022–2023 academic years for second-year students. Students whose application files were incomplete at N-1, those repeating their second year of medicine at N-1, and students admitted through a bridge program were not included.

The Faculty of Medicine at the University of Tours is the only medical faculty in the *Centre-Val de Loire* region (39,150 km² area; 2,574,863 inhabitants). In 2022, there were 350 doctors for every 100,000 inhabitants, representing one of the lowest medical densities in France [4]. The *Centre-Val de Loire* region comprises six areas, each with its own main administrative office: Tours, Blois, Orléans, Chartres, Châteauroux, and Bourges (see Supplemental material 1).

### Ethics

Permission to access data from the APOGEE (French universities database) was granted by the University of Tours. Only strictly necessary data were accessed, and it was extracted anonymously and used in compliance with both French and European legislation. This study was approved by the Institutional Ethics Committee of the University Hospital of Tours (n°2,023,060). According to French Law, informed written non-opposition was obtained from all subjects, ensuring compliance with ethical and regulatory standards.

### Data collection

For each student in the study, the following information was extracted from APOGEE database: date of birth, gender, N-1 year of PACES/PASS/L.AS, baccalaureate (results, specialization, and year), parents’ region of residence and socio-economic background (according to the National Bureau of Statistics, INSEE).

### Statistical analyses

To facilitate comparison between year levels, quantitative data was expressed in absolute values and qualitative in relative values (percentage in relation to the year). Data analysis was performed using “R”, software version 4.02. The class of 2021–2022 (i.e., the reform year) and the sum of the previous three years’ results were compared using the following methods: quantitative variables were analyzed with the Student’s t-test, and qualitative variables were analyzed using the Fisher test. Within the 2021–2022 year level, qualitative variables comparing the three entry streams (PACES, PASS, L.AS) were analyzed using the Chi-squared homogeneity test. The comparisons between the three entry streams for the entire study period were performed using One-Way Analysis of Variance (ANOVA) for quantitative variables and Chi-squared homogeneity tests for qualitative variables. A *p*-value < 0.05 was considered statistically significant.

## Results

**Fig. 1:**
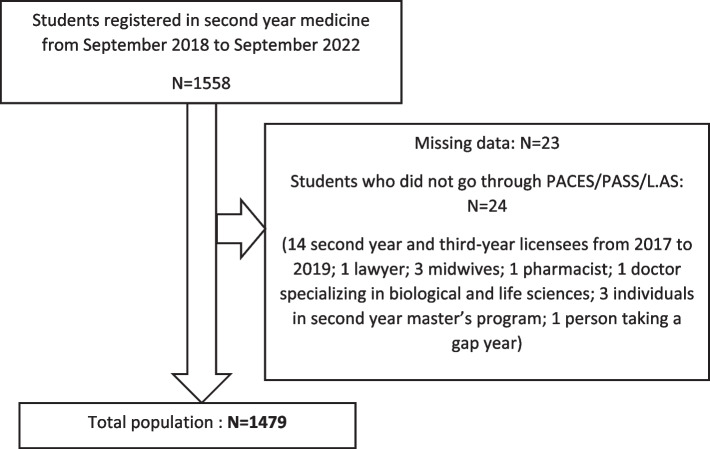
Flow diagram

The flow diagram is presented in Fig. 1.

### Comparison of the five year levels

The student population studied over the three periods is presented in Table 2. No difference was found in relation to average age nor gender ratio.

**Table 2:** Demographic data by year (NA: not applicable). In addition to the three-period analysis (pre-reform: 2018 to 2020; reform: 2021–2022; post-reform: 2022–2023), we present a comparison of baccalaureate results, geographic origin, and parental socioeconomic categories among students using the three entry pathways (PACES, PASS, L.AS) in Table 3 for the entire study period

	2018–2019	2019–2020	2020–2021	2021–2022	2022–2023
N students	259	269	279	337	335
Male (%)	83 (32.1)	110 (40.9)	89 (31.9)	117 (34.2)	102 (30.5)
Age (average, min-max, in years)	18 (16–19)	18 (16–33)	18 (16–30)	18 (16–34)	18 (16–34)
First Common year of Health Studies (PACES)	259	269	279	145	NA
Health Access Specific Pathway (PASS)	NA	NA	NA	136	193
Bachelor degree with Health Access Minor (L.AS)	NA	NA	NA	56	142

**Table 3:** Baccalaureate results, geographic origin and parental socioeconomic category for all PACES, PASS and L.AS students within the study period. ^1^n (%); mean (standard deviation). ^2^khi-squared test; One-Way Analysis of Variance (ANOVA). Significant *p*-values indicated in bold

	PACES (*N* = 952)^1^	PASS (*N* = 329)^1^	L.AS (*N* = 198)^1^	*p*-value^2^
Baccalaureate passed with high or highest honors	806 (85%)	317 (96%)	130 (66%)	**< 0.001**
Baccalaureate results (ordinal value)				**< 0.001**
Standard pass (0)	30 (3.2%)	3 (0.9%)	41 (21%)	
Honors (1)	113 (12%)	9 (2.7%)	26 (13%)	
High honors (2)	303 (32%)	83 (25%)	51 (26%)	
Highest honors (3)	503 (53%)	234 (71%)	79 (40%)	
Missing data	3	0	1	
Baccalaureate results (ordinal analysis)	2.35 (0.81)	2.67 (0.58)	1.85 (1.16)	**< 0.001**
Missing data	3	0	1	
Geographic origin				**< 0.001**
Tours (University City)	583 (61%)	153 (47%)	90 (45%)	
Neighboring areas within the Centre-Val de Loire region	353 (37%)	165 (50%)	87 (44%)	
Regions outside of the Centre-Val de Loire region	16 (2%)	11 (3%)	21 (11%)	
Number of parents belonging to white-collars and knowledge professional category				
0	346 (36%)	98 (30%)	85 (44%)	
1	345 (36%)	114 (35%)	70 (36%)	
2	261 (27%)	117 (36%)	37 (19%)	
Missing data	0	0	6	
Mean number of parents belonging to white-collars and knowledge professional category	0.911 (0.794)	1.058 (0.808)	0.750 (0.759)	**< 0.001**
Missing data	0	0	6	

#### Baccalaureate

During the reform year, fewer students obtained high honors or highest honors (147, 73.9%). In contrast, a greater number of students obtained honors and standard passes (89, 26.3%) compared to the average of the three previous years (Student’s t-test, *p* < 0.001, 95% CI [9.5; 20]) (Supplemental material 2).

One year after the reform, the figures returned to the same distribution as pre-reform years, with an increase in the number of the high honors and highest honors (292, 87.4%) and decrease among the honors and standard passes (42, 12.6%) (Supplemental material 2).

#### Geographic origins

**Fig. 2:**
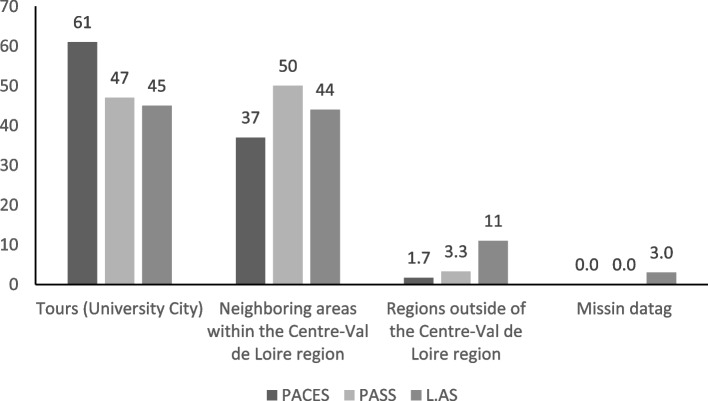
Breakdown of enrolled students based on parental domicile. The proportions of students originating from the six territories within the *Centre-Val de Loire* region for each entry stream are presented

Geographic origins of students based on their entry pathway are presented in Fig. 2.

During the reform year, there were proportionally fewer applicants from the Tours area (University City), with 51% compared to 64% during the average of the previous three years (*p* < 0.001, 95% CI [6.4; 20]). However, applications from neighboring areas and regions outside of the *Centre-Val de Loire* region increased (Supplemental material 3). This trend continued the following year with only 46.3% of the students coming from the Tours area (University City).

#### Parental socioeconomic category

**Fig. 3:**
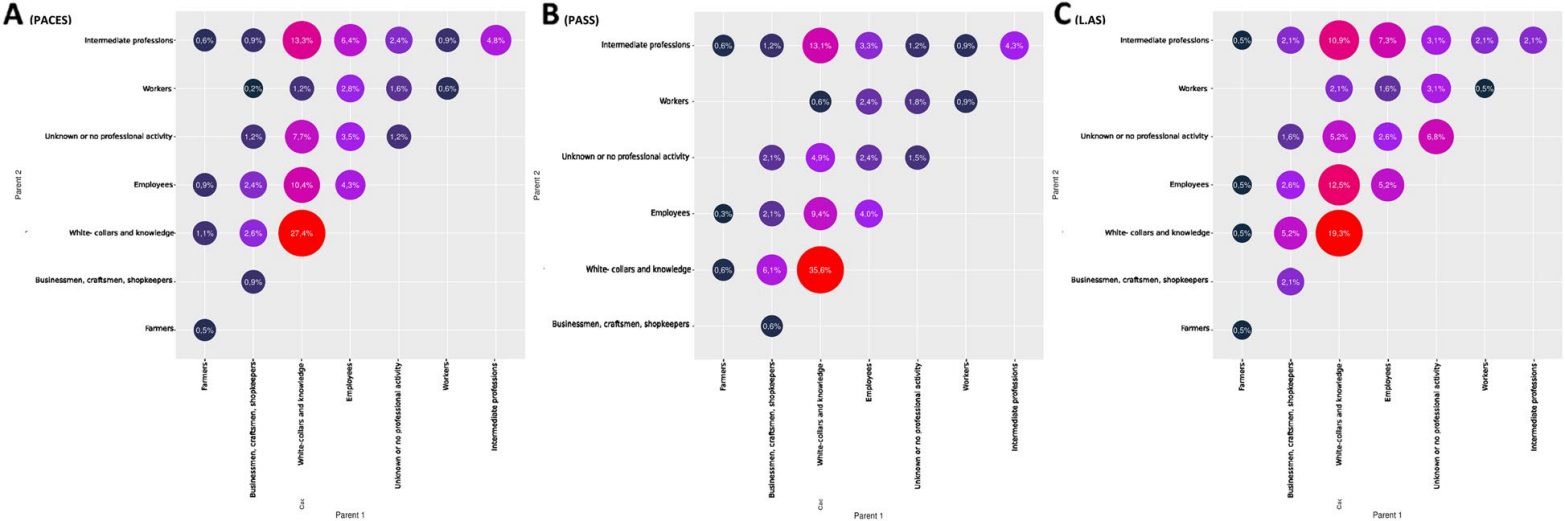
Parental socioeconomic category 1 and 2 according to the three entry pathways (A. PACES, B. PASS, C. L.AS)

In the whole cohort, two thirds of the students (64.0%) had one parent belonging to white-collars and knowledge professional category. One fourth of the students (28.1%) had both white-collar parents and knowledge professional category **(**Fig. 3**)**. This distribution remained stable among the five years.

### Results of the reform year (2021–2022)

#### Baccalaureate

The three entry streams presented differences in student baccalaureate levels. PACES and PASS streams resulted in a greater proportion of high honors and highest honors (respectively 64.6% and 95.6%) compared to L.AS (44.6%; Chi-squared homogeneity test, *p* < 0.001). The L.AS group included a higher proportion of students with a standard pass baccalaureate (44.6% compared to 8.3% in PACES and 1.5% in PASS).

#### Geographic origins

**Fig. 4:**
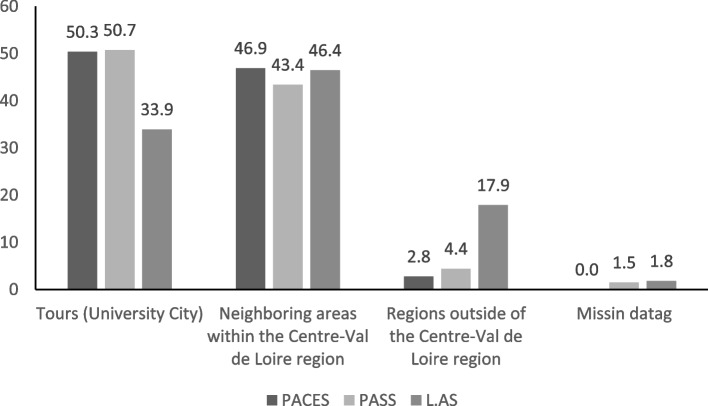
Graph of enrolled students in relation to their parental domicile for the reform year. The proportions of students originating from the six territories within the *Centre-Val de Loire* region for each entry stream (PACES/PASS/L.AS) are presented

 Half of the students came from the University area in PACES (50.3%) and PASS (50.7%) whereas one third only for L.AS (33.9%) (Fig. 4). This difference was statistically different when comparing PACES and PASS together versus L.AS (*p* = 0.023). The L.AS stream drew more student domiciled from territories outside of the *Centre-Val de Loire* region (17.9% compared to 2.8% in PACES and 4.4% in PASS).

#### Parental socioeconomic category

The proportion of students with at least one parent in the white-collar or knowledge professional category was higher in PASS (1 parent: 72.8% compared to 57.2% in PACES and 48.2% in L.AS (*p* = 0.002); 2 parents: 34.6% compared to 26.2% in PACES and 17.9% in L.AS (*p* = 0.051)).

### Results of the post-reform year (2022–2023)

#### Baccalaureate

2022–2023 was the first year following the reform year, without students from the PACES stream. Most students enrolled in PASS, had just passed their baccalaureate (187, 96.2%). In contrast those from L.AS had completed a one year diploma before L.AS (102, 71.8%).

Among the L.AS group, a greater number of students had standard pass or honors (25.5%) baccalaureate results compared to the PASS students (3.1%) where highest honors was more prevalent (70.5% compared to 48.2% in L.AS).

#### Geographic origins

The geographic origins between the two streams remained relatively homogenous, except in L.AS, where there was an increase in students from territories outside region *Centre-Val de Loire* (7.0% against 1.6% in PASS).

#### Parental socioeconomic background

The proportion of students with at least one or two white-collar parents and knowledge professional category was higher in **PASS** (1 parent: 56.5% compared to 47.9% in **L.AS**; 2 parents: 36.3% in **PASS** compared to 19.0% in **L.AS**).

## Discussion

The transition from the PACES selection mechanism to the PASS system marked a pivotal change. However, a significant increase in student diversity was predominantly observed in the L.AS pathway. This increase was relatively modest during the reform year, considering L.AS represented 17% of student enrollments into second year of medical studies. A year subsequent to the implementation of this reform, diversity among medical students improved as students originating from the L.AS pathway represented 42% of the total number of students.

The reform entry prerequisites stipulated for PASS, that the curriculum have a majority of health science subjects and an elective (non-health sciences) which would be chosen by the student, and inversely so for the L.AS. The aim of increasing student diversity through modifying the PACES selection process was not achieved. The PACES student profile (i.e., high honors or highest honors in their scientific baccalaureate results), was merely transferred to the PASS students. However, entry via the L.AS stream resulted in a new category of students with standard pass or honors for their scientific baccalaureate results. The following year, this diversity via the L.AS entry stream fell, and the L.AS became as competitive as the PASS.

In examining second-year medical students, it was noted that fewer had parents residing in the University City. The percentage declined from an average of 63% over the prior three years to 47%. This suggests an increased proportion of students originating from locations more distant from the university. Among the French medical faculties, the L.AS pathway curriculum varies depending on the resources available at each university. This curriculum heterogeneity could partly explain why students residing beyond the *Centre-Val de Loire* region, chose to apply outside their region. However, the openings provided by the L.AS stream to students coming from surrounding areas, diminished during the post-reform year.

It is crucial to acknowledge the potential for self-censorship among applicants. The PASS pathway, designed to replace PACES, may be perceived as especially competitive, whereas L.AS is viewed as an alternate entry route to medical studies. This perception of PASS’s heightened competitiveness might promote self-censorship tendencies among potential applicants. External factors, such as social background or the proximity of a student’s family home to the university, may also influence the selection process. A notable overrepresentation of students from the white-collar and knowledge professional sectors within PASS, compared to the general population ratio and in contrast to L.AS, lends credence to the hypothesis of student self-censorship during enrollment. Comprehensive research, inclusive of all first-year applicants and not just those who succeeded as in our current study, is necessary to ascertain the influence of these factors on medical student selection.

Parents enrolling their children into first year medicine, understandably, would wish to optimize their child’s chances for success. One could hypothesize that certain candidates believed that the less competitive L.AS stream, would increase their likelihood acceptance into medical studies. The proportional increase of higher scoring students in L.AS in the post-reform year supports this hypothesis. In addition, our observations would indicate that high school students strategically modify their performance and choices in relation to the selection system. This adaptive approach to course selection could in the end, render moot real diversification. In summary, the L.AS stream entry to second year medicine enabled access to a wider range of student profiles, albeit temporarily.

Concurrent with the pedagogic reform at the Medical Faculty of the University of Tours, there was a notable rise in the intake for medical studies. The intake increased by 22.2% (60 additional students) in 2021–2022, in contrast to a 3.7% rise (10 additional students) over the previous two years. In response to parental concerns about the first-year medical reform, the French Council of State mandated the Faculty of Medicine in Tours, along with 14 other such faculties in France, to admit a larger number of students into the second year of medicine [5]. The French Council of State recognized that seats in the second year of medical studies were largely allocated to students from the previous system, PACES, even if the intention was to ensure fairness for the new first-year students in the recently introduced courses, PASS and L.AS. Even though PACES students represented 30% of the first-year cohort, they were given nearly 48% of the positions in the second year. Additionally, the Council of State pointed out that fifteen universities had not sufficiently expanded their second year enrollment capacity. This posed a risk that the reform could disadvantage PASS and L.AS students.

There is growing scientific evidence that a diverse health care workforce will contribute to improve national health care for the population [6–8]. Medical schools must cultivate a broader applicant base that mirrors the demographic composition of the general population, ensuring a representative mix among medical students [9]. According to our findings, achieving a diverse health care workforce is still a distant objective, and its realization hinges on the inclusiveness of the national medical student population.

The observations of this study should take into account certain limitations, such as the non-inclusion of “transfer” students – who enter second year medicine via an alternative selection pathway who remain outside the average student profile. Nevertheless, the number of transfer students remains modest, averaging approximately ten students per academic year. Another limitation of this study lies in its monocentric nature. However, this approach is still more favorable than a multicentric comparison which could involve data from varied and inconsistent admission systems.

## Conclusion

The reform replaced the elite selection system PACES with two pathways PASS and L.AS. Increased student diversity was observed in the L.AS stream which represented 42% of the total number of students during the post-reform year. Certain universities suggested only maintaining the L.AS stream as the sole entry point to health education, in order to avoid PASS being a disguised PACES. It remains to be seen how these reform changes in student selection might influence the future doctors, their professional pathways, both geographical distribution and mode of practice, and ultimately the quality of care offered. These points should be followed up in the coming years.

### Supplementary Information

Supplementary Material 1. Areas constituting the *Centre-Val de Loire* region and their location in France. The logo represents the University of Tours.

Supplementary Material 2. Results in relation to the baccalaureate (time frame and level) (Due to *2022 baccalaureate reform, stream identification was unavailable).

Supplementary Material 3. Breakdown of enrolled students based on parental domicile. The proportions of students originating from the six territories within the *Centre-Val de Loire* region for each year are presented.

## Data Availability

The datasets used and/or analyzed during the current study are available from the corresponding author on reasonable request.
