# Supplementary figures and images for: Reform of the first year of medical studies and diversification of student profiles in France: an unmet need?
Source: BMC Med Educ. 2024 May 28;24:581. doi: 10.1186/s12909-024-05570-4 (PMC11134893; doi:10.1186/s12909-024-05570-4)

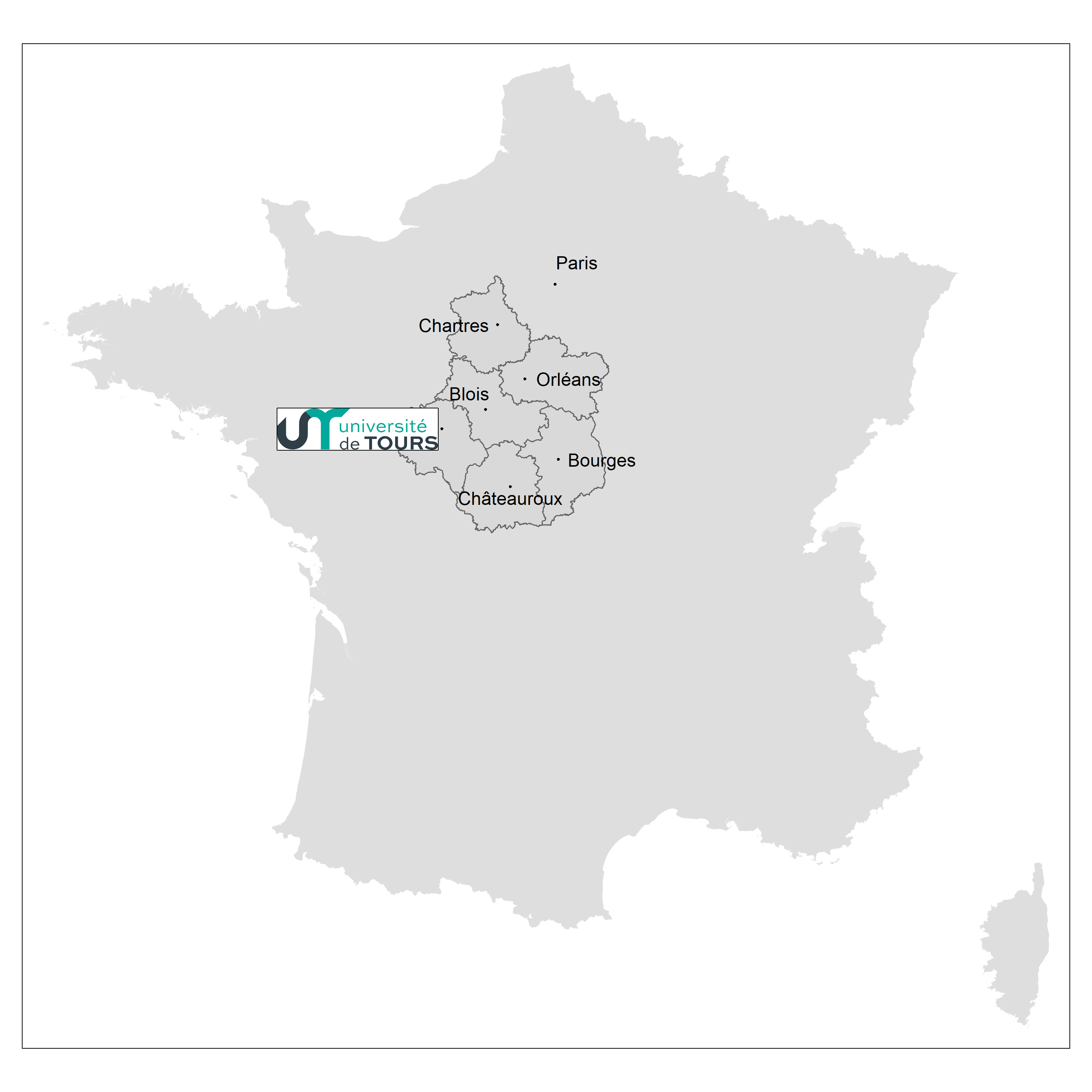

Supplement: Supplementary file 1 — Supplementary Material 1. Areas constituting the Centre-Val de Loire region and their location in France. The logo represents the University of Tours. [file 12909_2024_5570_MOESM1_ESM.png]
