# Supplementary material for: Reform of the first year of medical studies and diversification of student profiles in France: an unmet need?
Source: BMC Med Educ. 2024 May 28;24:581. doi: 10.1186/s12909-024-05570-4 (PMC11134893; doi:10.1186/s12909-024-05570-4)
Supplement: Supplementary file 2 — Supplementary Material 2. Results in relation to the baccalaureate (time frame and level) (Due to *2022 baccalaureate reform, stream identification was unavailable). [file 12909_2024_5570_MOESM2_ESM.docx]

**Supplemental material 2** Results in relation to the baccalaureate (time frame and level) (Due to *2022 baccalaureate reform, stream identification was unavailable).

|  | 2018-2019 | 2019-2020 | 2020-2021 | 2021-2022 | 2022-2023 |
| --- | --- | --- | --- | --- | --- |
| N students | 259 | 269 | 279 | 337 | 335 |
| N students who passed the first year after completing the baccalaureate (n, %) | 120 (46.3) | 117 (43.5) | 115 (41.1) | 142 (41.9) | 210 (62.7) |
| N students who passed the first year 1 year after the baccalaureate (n, %) | 134 (51.7) | 142 (52.8) | 155 (55.4) | 160 (47.2) | 108 (32.2) |
| N students who passed the first year ≥2 years after the baccalaureate (n, %) | 4 (1.5) | 9 (3.3) | 9 (3.2) | 36 (10.6) | 17 (5.1) |
| Ratio of scientific baccalaureate/total (%) | 99.6 | 99.3 | 98.6 | 98.8 | 36.7* |
| Other baccalaureate | 0 | 1 | 3 | 3 | 1 |
| Other degrees | 1 | 1 | 1 | 1 | 1 |
| Standard pass (n, %) | 6 (2.3) | 7 (2.6) | 5 (1.8) | 40 (11.6) | 17 (5.1) |
| Honors (n, %) | 28 (10.9) | 23 (8.6) | 24 (8.2) | 49 (14.6) | 25 (7.5) |
| High honors (n, %) | 79 (30.6) | 66 (24.6) | 102 (36.6) | 102 (30.4) | 88 (26.3) |
| Highest honors (n, %) | 145 (56.2) | 172 (64.2%) | 149 (53.4) | 147 (43.5) | 204 (61.1) |
