# Supplementary material for: Reform of the first year of medical studies and diversification of student profiles in France: an unmet need?
Source: BMC Med Educ. 2024 May 28;24:581. doi: 10.1186/s12909-024-05570-4 (PMC11134893; doi:10.1186/s12909-024-05570-4)
Supplement: Supplementary file 3 — Supplementary Material 3. Breakdown of enrolled students based on parental domicile. The proportions of students originating from the six territories within the Centre-Val de Loire region for each year are presented. [file 12909_2024_5570_MOESM3_ESM.docx]

**Supplemental material 3** Breakdown of enrolled students based on parental domicile. The proportions of students originating from the six territories within the *Centre-Val de Loire* region for each year are presented.
